# Last Aid Course. An Education For All Citizens and an Ingredient of Compassionate Communities

**DOI:** 10.3390/healthcare7010019

**Published:** 2019-01-28

**Authors:** Georg Bollig, Frans Brandt, Marius Ciurlionis, Boris Knopf

**Affiliations:** 1 Palliative Care Team, Medical Department Sønderborg/Tønder, South Jutland Hospital, 6400 Sønderborg, Denmark; Frans.Brandt.Kristensen@rsyd.dk; 2 Medical Research Unit, Institute of Regional Health Research, University of Southern Denmark, 6200 Aabenraa, Denmark; 3 Last Aid International, 24837 Schleswig, Germany; marius.ciurlionis@gmail.com (M.C.); boris.knopf@wuerdezentrum.de (B.K.)

**Keywords:** death, dying, home death, palliative care, Last Aid course, compassionate communities

## Abstract

Due to demographic changes, the need for palliative care in the community and at home is expected to rise in the coming years. The care that is given by family members and general practitioners plays a vital role in basic palliative care. Knowledge in palliative care is very limited or totally absent in most communities, and information about the effects of educational procedures in teaching non-professionals in basic palliative care is sparse. In the Last Aid course, the public knowledge approach and the initial experiences from the implementation process are described. In addition, a review of the literature on educational efforts regarding palliative care for non-professionals and the existing literature on Last Aid courses is provided. An international working group has established a curriculum for Last Aid courses based on four teaching hours (45 minutes each). The feasibility of Last Aid courses for the public has been tested in pilot courses. The experiences with Last Aid courses in different countries are overall very positive. Last Aid courses are well-attended. The evaluation of questionnaires in a German pilot study has shown a favorable response. Last Aid courses may form the educational basis of compassionate communities, and are well-suited to inform the public about palliative care and end-of-life care.

## 1. Introduction

The demand for palliative care in communities and at home is expected to rise in coming years. The main reasons for this trend are the demographic change with an increasing number of old people, an increasing incidence of chronic diseases, and an increasing number of people in need of palliative care. This expanded need for palliative care is in part caused by a raising awareness that patients with diseases other than cancer have palliative care needs as well, and should also have access to both general and specialized palliative care in the community. Experts say that the global demographic change will lead to an increase in the number of multi-morbid and frail elderly people [1,2]. Dying at home is a wish that many people have [3,4]. In a sample of European countries including England, Flanders, Germany, Italy, Netherland, Portugal, and Spain, two-thirds of people stated that they prefer a home death in all but one country [4]. Care by family members and general practitioners are important factors in strengthening basic palliative care in the community and in making home-death become a reality for more people [5]. So far, public education in palliative care is very limited or totally absent in most communities [6].

We provide an overview on the Last Aid course concept and the compassionate community approach. In addition, a short literature review on educational efforts regarding palliative care for non-professionals is provided as the basis for a discussion on the topic. 

## 2. Materials and Methods

This article is based on a review of the existing publications regarding Last Aid courses, our own experiences of their implementation, and the existing literature on palliative care education for citizens. In addition, a literature search with the MeSH (Medical Subject Heading) words “palliative care education”, “public”, “caregivers”, and “compassionate communities“, was performed using the search engines PubMed and Medline All publications that had public palliative care education as a major topic were included. A short overview of the existing literature on this topic is provided. 

## 3. Findings

The Last Aid course is a relatively new concept for teaching the public about palliative care. In the first parts of this section, the existing knowledge about palliative care education for the public and compassionate communities is summarized. Thereafter, the idea of Last Aid and its implementation in different countries is described.

An initial literature search in PubMed and Medline for the term “palliative care education” retrieved 405 papers. In contrast, the above described search strategy with a combination of all search terms (public, caregivers or compassionate communities, and palliative care education) lead to 47 papers with accessible abstracts. The authors have reviewed all abstracts. Of these 47 papers only 7 papers included palliative care education for the public as a major topic. Additional sources were found by hand searches in books and reference lists or the Internet. 

### 3.1. Palliative Care Education for the Public

The literature search identified 405 papers in PubMed and/or Medline. In contrast to the majority of these papers, which provide information on teaching professionals, few articles describe educational efforts in basic palliative care and end-of-life care for nonprofessionals. Lay people lack knowledge about palliative care [6], and there is an urgent need to educate nonprofessionals in palliative care and end-of-life care [7,8,9,10,11,12]. At present, a main source of citizens’ palliative care knowledge is personal experience [13]. Approximately half of informal home hospice caregivers have unmet information needs [14]. Topics that should be addressed are general information about hospice care including the level of support offered and what to expect at the end of life [14]. Unfortunately, most educational information material on palliative care that is available for the public on Google (i.e., on the Internet) does not meet the national literacy level in America [15]. Unfortunately, this proves to be a barrier in attaining knowledge about palliative care, especially for those without higher education. 

Nevertheless, there are examples of already-existing initiatives for the education of the public from different countries. Family carers in Australia have been educated in three successive weekly group sessions [16,17]. The psycho-educational group education was found to be useful in preparing citizens in providing support for dying relatives at home [17]. There are also some other more informal concepts on educating nonprofessionals in discussing death and dying. These include initiatives such as the “death cafe” [18] and the “death chat” at St. Christopher’s hospice in London [19], among others. The lack of death literacy is a common problem in many countries. Death literacy consists of four parts:1) skills, 2) knowledge, 3) experiential learning, and 4) social action. It is not enough to only talk about death—social action is needed [20]. This underlines the fact that education alone is not the solution in improving palliative care in the community. Education must be accompanied by a reflection on attitudes, as well as action. Without reflection and action, there may be no change in practice and no practical improvement.

### 3.2. Compassionate Communities

Compassionate communities are examples of the engagement of neighborhoods in caring for others as a humanitarian practice, which includes palliative care and end-of-life care provision. Kellehear was the first to introduce the term “compassionate community”. He stated that compassionate communities are needed as a public health approach to palliative care [21,22]. Kellehear also called all citizens to action by his statement: “end-of-life care is everyone’s responsibility” [21]. Librada Flores et al. have introduced a systematic approach called “All with you” for the development of compassionate communities that has been used in cities in Spain and South America [23]. This approach is based on three factors: social awareness, training, and the implementation of networks. Important success criteria for its implementation were, among others, strong leadership and a high participation rate [24]. A barrier for the implementation of compassionate communities may be the reluctance of carers to ask for, and to accept, help from others [25]. It has been demonstrated that laymen are capable of participating in palliative care provision. In Poland, prisoners participate in end-of-life care in the community as hospice volunteers [26]. The cooperation of citizens and interprofessional healthcare workers can enable the continuum of compassionate palliative care [25]. The inclusion of death education in the school curriculum may be one method of raising public awareness as part of the implementation process to build a compassionate community [27]. In Germany, a program for a school project week called “Hospiz macht Schule” about death and dying is used to educate school children [28]. It has been recommended that the implementation of a compassionate community approach should include a community education program and encompass death education in schools [29]. McLoughlin suggests running courses for the public over 8 to 12 weeks with one weekly session, and furthermore, that the following topics should be included [29]:

- Attitudes toward death, dying, loss and care;
- Social, cultural, and historical influences on death and dying;
- Mortality and society;
- Death and dying in healthcare;
- End-of-life issues and decisions;
- Facing death—living with life-threatening illness;
- Last rites: funerals and body disposition;
- Death in the lives of children and adolescents;
- Death in the lives of adults;
- Beyond death/after life;
- The path ahead: personal and social choices.

### 3.3. Development and Implementation of Last Aid Courses

The idea of Last Aid courses and a public knowledge approach to palliative care were first described by Bollig in his master’s thesis at the University of Klagenfurt/IFF Vienna, Austria in 2008. These ideas were then presented to an international audience during the congress of the European Association for Palliative Care (EAPC) in Vienna in 2009 [30], and the master’s thesis was published as a book in 2010 [8]. Between 2009 and 2011, a course curriculum of a Last Aid course was discussed in a working group with members of the Austrian Red Cross and the IFF Vienna, University Klagenfurt. It included the same amount of teaching hours as a first aid course for citizens (i.e., 16). Although much effort was used to establish the concept, it has not yet been put into practice [31]. Some years later, a working group from Norway, Denmark, and Germany established a Last Aid course curriculum between 2013 and 2014 with four teaching hours based on the initial idea and recommendations by Bollig [8]. The four modules of the Last Aid course cover the following themes: care at the end of life, advance care planning and decision making, symptom management, and cultural aspects of death and bereavement (Table A1). Usually, the Last Aid course is held on one single day (afternoon or evening) with four teaching units of 45 minutes each. The course is then divided into two parts with 1.5 hours and a 30-minute break in between. The first Last Aid courses were held from the end of 2014 in Norway and 2015 in Germany and Denmark [32,33,34,35,36,37,38,39].

**Figure A1:**
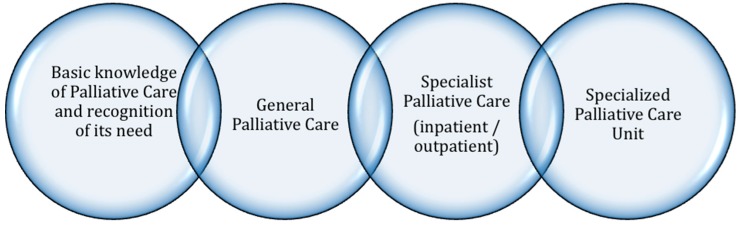
The Chain of Palliative Care (from Bollig 2010) [8].

The Last Aid concept is built on the presupposition that palliative care knowledge should become a part of public education. The chain of palliative care shows the cooperation of both nonprofessionals and health care professionals in palliative care in order to meet the needs of the patient and their relatives (Figure A1). Similar to the chain of survival in emergency medicine, a “Chain of Palliative Care” is presented to illustrate palliative care pathways in the community [8]. The public knowledge approach [8] aims at introducing palliative care knowledge in the entire society in order to enhance public awareness, reflection, and discussion in various settings. Education about death and dying should be a part of the school curriculum. In the future, the public knowledge approach, including the widespread education of all citizens, may contribute to changing attitudes and behaviors in the community towards a more positive attitude in participating in palliative care. 

The Last Aid project in Germany has achieved an award from Startsocial (a German initiative to support and promote social projects) and chancellor Angela Merkel [38] for being one of the best social projects in Germany in 2015. The project received a prize from the German Association for Palliative Medicine in September 2015 for a pilot study on the first German Last Aid courses in Schleswig in Northern Germany [34,35]. An initial mini review on the Last Aid course was published in 2016 [35]. In May 2017, the first German Last Aid Symposium was held in Hamburg. The second German Last Aid Symposium took place in Kassel in October 2018, where more than 90 participants discussed the Last Aid course concepts, including actual practice and ideas for future improvement. Both meetings were supported by the Paula Kubitscheck-Vogel-Stiftung in Munich, Germany. In Germany, Last Aid courses are recognized and recommended as basic education in palliative care for both citizens and health care professionals by the German Hospice Association (Deutscher Hospiz und Palliativvverband-DHPV) and the German Association for Palliative Medicine (Deutsche Gesellschaft für Palliativmedizin-DGP). The Last Aid course has been presented during a number of national and international conferences, among others in Montreal in 2016 and Edinburgh in 2017 [36,39,40,41,42,43]. Some of the lectures are available on YouTube. The first international Last Aid congress is planned to be held in Denmark in autumn 2019. 

The curriculum and the slide presentation of the Last Aid course was evaluated and adapted by the International Last Aid working group in May 2018 (see Table A1). During the meeting, a consensus on some minor revisions and changes was achieved. At present, the international working group consists of members from the following countries:

- Germany;
- Denmark;
- Austria;
- Switzerland;
- Lithuania;
- Slovenia;
- Scotland;
- Estonia;
- Latvia.

Further expansion of the International Last Aid working group is planned, and at present several other countries are in the process of joining the group. In 2016, a small handbook on Last Aid (“Letzte Hilfe”) for course participants was published in German [44]. A Danish handbook, with the title “Sidstehjælp”, was published in October 2018 [45]. Books in other languages are planned.

The experiences with Last Aid courses in different countries are overall very positive. People appreciate the course and recommend it to others. There is a high demand for courses by the public, and instructor courses are held with regular intervals. As of December 2018, more than 8500 citizens have participated in Last Aid courses and over 1000 Last Aid course instructors have been trained. All course participants are asked to fill in a questionnaire in order to evaluate the participants’ view. In addition, Last Aid course instructors provide their views in focus group interviews. The scientific evaluation is ongoing. The initial evaluation from Germany has shown that talking about death and dying was appreciated by the majority of participants. Accordingly, a high proportion of participants would recommend the course to others. In a German pilot study from 2015, a questionnaire about the Last Aid course was used as an evaluation tool. All participants stated that they would recommend the course to others [34]. Examples of statements from the German course participants are [34]:

- If I only had known that before it would have helped me when my aunt died.
- I appreciate the natural way to deal with the topics death and dying.
- Lively and easy although the topic is complicated.
- I have no suggestion that could improve the course.
- Clear and structured.

A combination of Last Aid courses and compassionate communities may improve end-of-life care for people dying at home. Last Aid courses can both provide basic education for citizens while simultaneously providing an arena in which to discuss and reflect on death and dying in the community.

## 4. Discussion

The main findings from our literature review and the experiences with the implementation of Last Aid courses are: that the participation of citizens is important to improve palliative care at home due to the limited resources for specialized palliative care at home and an anticipated high number of future home deaths; and that the public lacks information about palliative care and end-of-life care in general. Therefore, the education of the public in palliative care and in end-of-life care is needed. 

Many Last Aid course participants share their experiences by contacting Last Aid International or local instructors. As a result of course participation, people often talk about death, dying, and end-of-life care with their family, friends, and colleagues after the course. Many participants reported that they used the new knowledge after the course. Unfortunately, a participant became seriously ill from cancer shortly after the course and contacted the palliative care team of the hospital whose members had previously taught the Last Aid course that she attended. The patient’s cancer was incurable; she lived alone and therefore wanted to plan palliative care and death at home (personal communications). Thus, she illustrated her use of newly attained knowledge.

There is a definitive need for caring communities and the participation of citizens in the provision of palliative care and end-of-life care [21,22]. In order to enable citizens to care for seriously ill and dying people, there is a need for awareness, education, reflection, and public discussion [8,17,18,46]. Singer has pointed out that information about end-of-life care needs to address the public [9]: “Today, the challenge is to develop systematic and comprehensive information on the quality of end of life care at the population level.”

Education should be as short as possible [47]. The Last Aid course concept addresses the suggestions made by Singer and Ferris and provides information for the public in the shortest time possible. It uses a one-time education model, with four modules of 45 minutes each which are usually taught on a single afternoon or evening. Last Aid courses contain important features from a psychological viewpoint. They are important as educational initiatives for the public, may strengthen the self-help ability of citizens, and may lead to the empowerment of the local community in facing death and dying [48].

The EAPC-whitepaper on education in palliative care has defined three levels of education that include: 1. Palliative care approach; 2. General palliative care; 3. Specialist palliative care [49]. The Palliative care approach is defined as follows: A way to integrate palliative care methods and procedures in settings not specialized in palliative care. This should be made available to general practitioners and staff in general hospitals, as well as to nursing services and nursing home staff. This may be taught through undergraduate learning or through continuing professional development [49].

The three steps described above are aimed at health care professionals, while the public knowledge approach described by Bollig [8] can be seen as a basic educational level for the public. It aims to integrate the whole society in palliative care provision, as suggested by Kellehear [21,22], while simultaneously focusing on the education of the general public in the shortest time possible [47,48]. Thus, the public knowledge approach has a large potential to inform wide parts of the population. It may help to enhance both public discussion and the formation of a higher number of compassionate communities. This combined approach could improve both the knowledge and skills of the public, and lead to experiential learning and social action. This in turn could contribute to increasing death literacy. 

**Figure A2:**
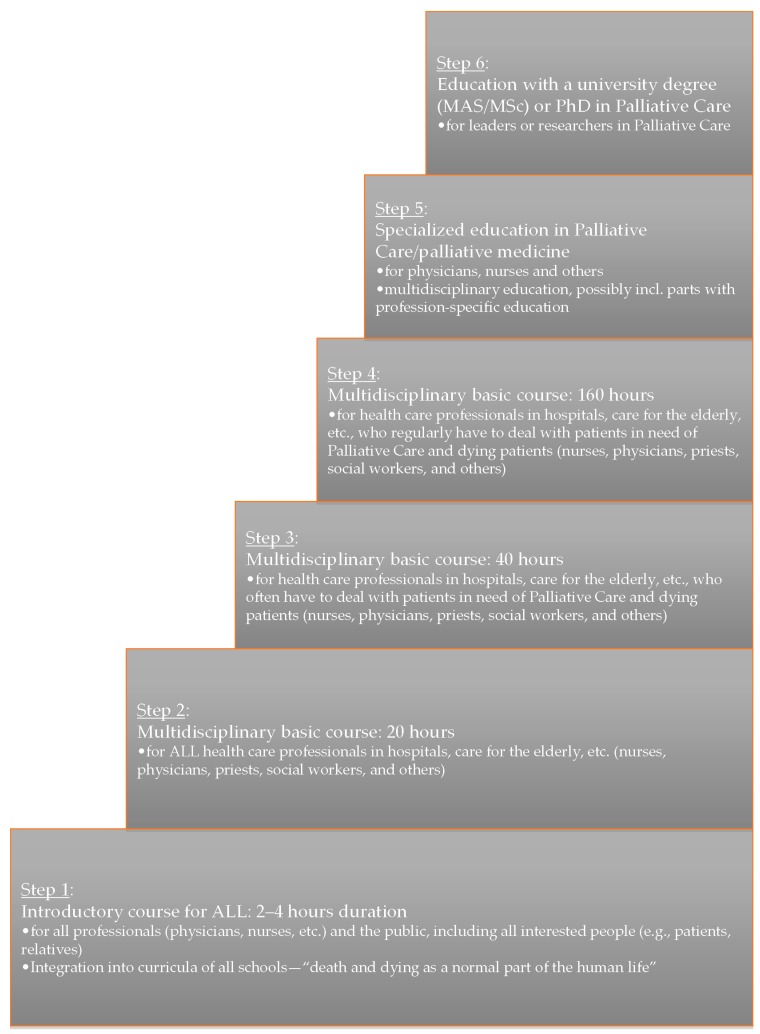
The six-step approach to education in Palliative Care (from Bollig 2009) [52].

All ten of the core competencies described by the EAPC [49] are included in the Last Aid course curriculum on a basic level that is suitable for the public. The six-step model of learning in palliative cares (see Figure A2) incorporates all citizens, and underlines that learning in palliative care, as a lifelong process, starts in school [50]. Kellehear highlights that it is every citizen’s responsibility to provide end-of-life care [21]. This approach aims to enable citizens to care for each other and to participate in end-of-life care provision, and is similar to the public knowledge approach [8,30,43]. Other communication channels, such as mass media including television and the Internet, could help to educate the public about palliative care in the future. In order to educate citizens with different educational levels, it is important to use language that can be understood by ordinary people [15]. In the Last Aid course, the instructors focus strongly on the use of everyday language to educate the public and to avoid language that is “too” professional. Probably in the future, drama may even play a role in palliative care education for citizens [51]. 

As described above, there is an urgent need to educate citizens in palliative care and end-of-life care, including advance care planning. Education in local communities, even engaging teenagers, is possible and may lead to their involvement, participation, and empowerment [43]. The “compassionate communities” approach, the “public knowledge” approach with Last Aid courses, and other approaches (e.g., the death chat or death cafe [18,19] discussed above, are the most promising efforts to teach the public about palliative care. Probably, these efforts could be combined in the future. A combination of Last Aid courses with the compassionate community approach may help to increase the participation of citizens in palliative care and end-of-life care. This may help to increase the number of home-deaths for many people who would prefer to die at home. Last Aid courses can therefore be seen as an important ingredient of compassionate communities, and may even be seen as their educational foundation. From 2017–2019, the international dissemination of Last Aid courses is a project titled “Last Aid International—The Last Aid Movement“, in connection with the European Palliative Care Leadership Academy [52]. The preliminary results on the use of the Last Aid course as basic palliative care education for non-medical personnel in a university hospital indicate that this approach is feasible and useful [53]. A Last Aid course for children aged 8–14 years has also been pilot-tested successfully [54].

## 5. Conclusions

The public requires more information and education about palliative care. The Last Aid courses are accepted by many and have an enormous potential to spread information about palliative care throughout the public and can enhance public discussion about end-of-life. The experience of teaching the public in Last Aid courses in different countries and in different arenas has been encouraging. Doing research on the education of nonprofessionals in palliative care, end-of-life care, and the compassionate community approach is necessary. Research on the implementation of the Last Aid course is currently being undertaken in different countries. A combination of the Last Aid course concept and the compassionate community approach may together comprise the greatest potential to improve palliative care at home for many people. 

## Appendix A

**Table A1:** The Last Aid course contents (version May 2018). Last Aid Care for seriously ill and dying people at the end of life.

Module nr.	Topic	Course Content
Module 1	Dying as a normal part of life	Welcome and introductions First Aid and Last Aid What you can do to care The process of dying
Module 2	Planning ahead	Networks of Support Making decisions Medical and ethical aspects Advance care planning Advance Directive Medical Power of Attorney
Module 3	Relieving suffering	Typical problems and symptoms Caring/relieving suffering Nutrition at the end of life How to comfort
Module 4	Final goodbyes	Saying good bye/final fare-well rituals Funeral and various forms of burials Grieving is normal Grief and ways of grieving Questions, Comments
